# Impact of hypertension and lipid profile on cardiac function in an Iranian teaching hospital: A cross-sectional analysis

**DOI:** 10.21542/gcsp.2024.29

**Published:** 2024-08-01

**Authors:** Fatemeh Omidi, Mohammad Javad Nasiri, Soheila Sadeghi

**Affiliations:** 1 Department of Cardiology, Imam Hossein Hospital, Shahid Beheshti University of Medical Sciences, Tehran, Iran; 2 School of Medicine, Shahid Beheshti University of Medical Sciences, Tehran, Iran; 3 Department of Internal Medicine, Imam Hossein Hospital, Shahid Beheshti University of Medical Sciences, Tehran, Iran

## Abstract

Introduction: Cardiovascular diseases (CVDs) pose a significant global health challenge and affect diverse populations. For conditions such as coronary artery disease, heart failure, and stroke prevalence, understanding the multifaceted factors contributing to CVDs is crucial. Hypertension and dyslipidemia, which are established risk factors, require further exploration, particularly in terms of their individual impact on cardiac function. This study aims to uncover associations, elucidate nuanced interplay, and provide insights for personalized interventions.

Methods: A cross-sectional analysis, conducted at a Tehran Teaching Hospital, focused on adults admitted for suspected heart disease. The dataset included demographic information, clinical history, medications, and echocardiographic data. Statistical analysis was performed using Pearson’s correlation with IBM SPSS Statistics software.

Results: In this analysis of 95 individuals suspected of heart disease, aged 51 years on average, diverse blood pressure patterns with significant percentages across various hypertension stages were observed. Lipid profile analysis revealed typical lipid levels. Correlations between blood pressure, lipid parameters, and cardiac function indicated significant associations, including a negative correlation between ejection fraction and blood pressure and significant correlations between lipid profiles and adverse cardiac volume changes. Disparities in the prevalence of obesity and diabetes have highlighted potential links to hypertension.

Conclusions: This study sheds light on crucial clinical aspects of individuals with suspected heart disease, revealing patterns of obesity, varied blood pressure categories, and nuanced lipid profiles. The correlations between blood pressure, lipid parameters, and cardiac function highlight potential connections, emphasizing the importance of tailored interventions for improved cardiovascular outcomes.

## Introduction

Cardiovascular diseases (CVDs) are a formidable global health challenge that impose a significant burden on both public health resources and individuals worldwide^1^. The impact of CVDs extends across diverse populations, affecting people with varying demographics and socioeconomic backgrounds^1,2^. This health challenge is characterized by the prevalence of conditions such as coronary artery disease, heart failure, and stroke, which collectively contribute to substantial morbidity and mortality rates^3,4^. The multifaceted nature of CVDs necessitates a comprehensive and concerted effort to understand their intricate etiology, risk factors, and underlying mechanisms driving their progression^4,5^. Among well-established and modifiable risk factors, hypertension and dyslipidemia are major contributors to the development and exacerbation of CVDs^5–9^. Existing research has independently linked hypertension and dyslipidemia to adverse cardiovascular outcomes^10–15^. However, the specific interplay between these factors, particularly their individual effects on cardiac function, remains an area that requires thorough investigation. Understanding how hypertension and dyslipidemia independently influence cardiac health is crucial for the development of targeted interventions and refining risk assessments. This study aims to investigate the associations between hypertension, lipid profiles, and cardiac function, offering insights that can inform personalized interventions and risk assessments. By highlighting these associations, this research not only contributes to existing academic knowledge, but also seeks to identify novel aspects that could potentially enhance strategies for improving patient outcomes in cardiovascular health.

## Methods

### Study design and participants

This one-year cross-sectional study, conducted at a Teaching Hospital in Tehran, Iran, focused on adult participants admitted with suspected heart diseases for selective coronary angiography. The decision to perform this procedure was made by the attending physicians based on noninvasive assessments.

To mitigate confounding factors, the study excluded individuals with a history of cardiac procedures, non-sinus rhythms, arrhythmias, liver or thyroid issues, diabetes, hypertension, elevated creatinine levels, inadequate echocardiographic views, ejection fraction <50%, valve stenosis, and moderate or severe valvular insufficiency. While these exclusions aimed to enhance internal validity, they may have limited the generalizability of the findings.

This study was approved by the Ethics Committee of the Shahid Beheshti University of Medical Sciences, Tehran, Iran (IR.SBMU.RETECH.REC.1402.659).

### Data collection

Demographic, physiological, and clinical information, including age, sex, height, weight, blood pressure, heart rate, lipid profiles, electrocardiogram (ECG), current medical history, and the presence of classic risk factors for cardiovascular diseases (including dyslipidemia, smoking, and triad) and medications (including lipid-lowering drugs, cardiovascular drugs such as beta-blockers, calcium channel blockers, angiotensin-converting enzyme inhibitors or angiotensin receptor blockers, aspirin, clopidogrel, and other similar drugs, diuretics, and nitrates), were collected from each patient upon their initial visit. Subsequently, the patients underwent transthoracic echocardiography to measure cardiac components and assess their heart function and structure.

### Statistical analysis

Pearson’s correlation analysis was used to investigate potential associations between hypertension, lipid profile, and cardiac function, assuming linear relationships. Pearson’s correlation was chosen for its ability to quantify the strength and direction of linear relationships between continuous variables, providing insights into how changes in one variable may relate to changes in another. However, it is important to note that the Pearson correlation may not capture nonlinear relationships that could exist among the variables studied. The significance threshold was set at *p* < 0.05. Statistical analyses were performed using IBM SPSS Statistics for Windows version 26 (IBM Corp).

## Results

### Participant characteristics

This cross-sectional study included 95 individuals with suspected heart disease, with a mean age of 51 years. The entire cohort exhibited a shared characteristic of obesity, as reflected by an average body mass index (BMI) of 27.8. Notably, clinical assessments revealed elevated blood pressure levels, with mean systolic and diastolic readings of 147 and 104 mmHg, respectively (Table 1). Remarkably, there was an absence of hyperlipidemia as well as a noteworthy lack of a family history of cardiovascular issues, coronary surgery, or related procedures among the participants. The metabolic profiles of the individuals, including fasting blood sugar, urea, creatinine, triglycerides, cholesterol, and high-density lipoprotein (HDL) levels, were within typical ranges. Hematological values, including hemoglobin and platelet counts, were also within normal limits.

### Prevalence of hypertension

The results of blood pressure categorization in our study cohort, based on the American College of Cardiology (ACC) and American Heart Association (AHA) guidelines, revealed diverse patterns. Approximately one-third of the individuals exhibited normal blood pressure levels, whereas 16.2% had elevated readings. A significant proportion, 26.2%, fell into Hypertension Stage 1, and 20.0% were classified under Hypertension Stage 2. Notably, 5.0% of the participants had experienced a hypertensive crisis.

### Lipid profile analysis

Table 2 shows the mean lipid profile of the study participants. The average total cholesterol level was 132.82 ± 49.76 mg/dL, with triglycerides at 151.53 ± 47.48 mg/dL. HDL cholesterol had a mean value of 44.98 ± 14.76 mg/dL, while low-density lipoprotein (LDL) cholesterol was recorded at 94.88 ± 32.21 mg/dL. These results provide insights into the lipid metabolism of the cohort and form a basis for assessing cardiovascular risk and health.

**Table 1:** Distribution of blood pressure levels.

**Blood pressure category**	**Frequency (%)**
Normal	32.5
Elevated	16.2
Hypertension stage 1	26.2
Hypertension stage 2	20.0
Hypertensive crisis	5.0

**Table 2:** Mean lipid profile values.

**Lipid parameter**	**Mean ± SD (mg/dL)**
Total cholesterol	132.82 ± 49.76
Triglycerides	151.53 ± 47.48
HDL	44.98 ± 14.76
LDL	94.88 32.21

**Notes.**

HDL High-Density Lipoprotein LDL Low-Density Lipoprotein

### Cardiovascular parameters

Table 3 shows the correlations among blood pressure, lipid parameters, and cardiac function in individuals with suspected heart disease. BP and DBP were strongly correlated (*r* = 0.67, *p* < 0.01). TG was positively correlated with Chol (*r* = 0.31, *p* < 0.01) and negatively correlated with HDL (*r* = −0.32, *p* < 0.01). Chol levels were strongly correlated with LDL levels (*r* = 0.87, *p* < 0.01). HDL was negatively correlated with LDL (*r* = −0.20, *p* < 0.05) and EDV (*r* = −0.26, *p* < 0.01). EF was negatively correlated with ESV (*r* = −0.53, *p* < 0.01), and EDV was positively correlated with ESV (*r* = 0.91, *p* < 0.01).

**Table 3:** Correlation matrix for cardiovascular parameters.

	**BP**	**DBP**	**TG**	**Chol**	**HDL**	**LDL**	**Cardiac function**		
							**EF**	**EDV**	**ESV**
**BP**	1	.67^**^	−.05	-.08	−.01	.00	−.01	.08	.07
**TG**	−.05	.06	1	.31^**^	-.32^**^	.22^*^	.01	−.12	-.12
**Chol**	−.08	−.03	.31^**^	1	.28^**^	.87^**^	.02	−.19	−.18
**HDL**	−.01	−.22^*^	−.32^**^	.28^**^	1	.03	−.05	−.26^**^	−.20^*^
**LDL**	.00	.13	.22^*^	.87^**^	.03	1	.00	−.09	-.08
**EF**	−.01	−.04	.01	.02	−.05	.00	1	−.19	−.53^**^
**EDV**	.08	.10	−.12	−.19	−.26^**^	−.09	−.19	1	.91^**^
**ESV**	.07	.10	−.12	−.18	−.20^*^	−.08	−.53^**^	.91^**^	1

**Notes.**

BP Blood Pressure DBP Diastolic Blood Pressure TG Triglycerides Chol Total Cholesterol HDL High-Density Lipoprotein LDL LowDensity Lipoprotein EF Cardiac Function, Ejection Fraction EDV End-Diastolic Volume ESV End-Systolic Volume

** Correlation is significant at the 0.01 level (2-tailed).

* Correlation is significant at the 0.05 level (2-tailed).

A statistically significant negative correlation was observed between the ejection fraction (EF) and blood pressure (*p* < 0.01), suggesting that higher blood pressure levels may be associated with adverse changes in cardiac pumping efficiency. Furthermore, the relationships between blood pressure and cardiac volume, specifically end-diastolic volume (EDV) and end-systolic volume (ESV), are complex. Statistical significance varies across parameters, suggesting a nuanced connection between blood pressure and cardiac volume.

In the context of lipid profiles, there were significant correlations between lipid profiles and cardiac function parameters, specifically total cholesterol and HDL. Total cholesterol was significantly positively correlated with ESV and EDV (*P* < 0.05). These findings suggest that higher cholesterol levels may be associated with adverse cardiac volume changes during both systolic and diastolic phases. HDL showed a statistically significant negative correlation with ESV and a trend of negative correlation with EDV (*p* < 0.05). This implies that lower HDL levels may be linked to adverse changes in cardiac volume during both phases of the cardiac cycle.

### Association with other risk factors

Table 4 illustrates the notable disparity in the prevalence of obesity and diabetes between the hypertensive and normotensive groups. The hypertensive group exhibited a higher obesity rate (55.5%) than of that the normotensive group (42.8%). Additionally, diabetes is more prevalent in the hypertensive group, with a percentage of 25.9%, compared to 17.1% in the normotensive group. These findings underscore the potential association between hypertension and elevated obesity and diabetes in the study population.

**Table 4:** Prevalence of other risk factors.

**Risk factor**	**Hypertensive group (%)**	**Normotensive group (%)**
Obesity	55.5	42.8
Diabetes	25.9	17.1

## Discussion

The principal findings of this study include the identification of significant correlations between blood pressure levels and cardiac function parameters. The observed negative correlation between EF and blood pressure suggests a potential link between higher blood pressure and adverse changes in cardiac pumping efficiency, which could influence cardiovascular management strategies. Additionally, the complex relationships between blood pressure and cardiac volumes, such as EDV and ESV, highlight the need for a nuanced understanding of these interactions. This study also revealed significant associations between lipid profiles and cardiac function, particularly Total Cholesterol and HDL, providing valuable insights into the intricate connections between lipid metabolism and cardiovascular health. These findings contribute to the evolving landscape of medical knowledge, emphasizing the importance of a holistic approach in addressing cardiovascular risk factors.

### Comparison with previous studies

Our findings on the significant correlations between blood pressure levels and cardiac function parameters are consistent with those of previous studies that have also observed similar associations. For example, studies by Li et al. (2023) and Sun et al. (2023) highlighted the impact of hypertension on adverse cardiovascular outcomes, supporting our observation of a negative correlation between ejection fraction and blood pressure^1,16^. Similarly, the correlations between lipid profiles and cardiac function observed in our study were consistent with the results reported by Hedayatnia et al. (2020) and Kaze et al. (2021)^11,12^. These studies also found significant associations between dyslipidemia and adverse changes in cardiac volume and function, corroborating our findings. However, our study provides additional insights into the nuanced interplay between these risk factors in an Iranian population, which has been less frequently studied than in Western populations. This highlights the importance of considering the regional differences in cardiovascular risk profiles and interventions.

While our results are largely in agreement with the existing literature, it is essential to note the unique aspects of our cohort, such as the absence of hyperlipidemia and a lack of a family history of cardiovascular issues, which may influence the generalizability of the findings. Further research on diverse populations is necessary to validate and expand upon these observations.

### Clinical implications

The clinical implications of this study are noteworthy for healthcare practitioners and policymakers. The identified correlations between blood pressure and cardiac function parameters underscore the importance of personalized cardiovascular interventions^17–21^. Clinicians can use these insights to tailor treatment strategies based on individual blood pressure profiles, potentially improving cardiac outcomes. Additionally, the observed associations between lipid profiles and cardiac function, specifically total cholesterol and HDL, suggest the need for comprehensive lipid management in cardiovascular care. Integrating these findings into clinical practice can guide health policy decisions by emphasizing preventive measures and early interventions targeting blood pressure and lipid abnormalities. This holistic approach may contribute to more effective strategies for reducing cardiovascular risk and enhancing overall heart health in patient populations^22–25^.

## Limitations

While this cross-sectional study provides valuable insights into the characteristics and associations within the study population, certain limitations should be acknowledged. First, the cross-sectional design inherently limits the establishment of causal relationships; therefore, caution is warranted when inferring cause-and-effect associations from the observed correlations. Additionally, the study cohort, consisting of individuals with suspected heart disease, may not be representative of the general population, which limits the generalizability of the findings. Moreover, reliance on self-reported data and potential recall bias can introduce inaccuracies in variables such as medical history and lifestyle factors. The potential impact of medication use (*e.g.*, lipid-lowering drugs and antihypertensives) on the study variables and interpretation of the results should also be considered. Further research with a longitudinal design and diverse participant cohorts is warranted to validate and extend the implications of these findings.

## Conclusions

This cross-sectional study reveals important clinical insights into individuals with suspected heart disease, including patterns of obesity, diverse blood pressure categories, and nuanced lipid profiles. The correlations between blood pressure, lipid parameters, and cardiac function underscore potential connections, emphasizing the importance of personalized interventions to enhance cardiovascular outcomes. The observed associations among obesity, diabetes, and hypertension underscore the need for comprehensive management strategies. Despite its valuable insights, further research is necessary to validate and deepen our understanding of these complex relationships.

## Competing interests

None.

## Data availability

The data used to support the findings of this study have been included in this article.

## Author contribution

All authors contributed equally to this work.
